# Significantly lower intramuscular pressure in the posterior and lateral compartments compared with the anterior compartment suggests alterations of the diagnostic criteria for chronic exertional compartment syndrome in the lower leg

**DOI:** 10.1007/s00167-020-06143-w

**Published:** 2020-07-08

**Authors:** Sophia Lindorsson, Qiuxia Zhang, Helena Brisby, Kajsa Rennerfelt

**Affiliations:** 1 Department of Orthopaedics, Institute of Clinical Sciences, Sahlgrenska Academy, University of Gothenburg, Gothenburg, Sweden; 2 Department of Orthopaedics, Sahlgrenska University Hospital, Gothenburg, Ortopedmottagningen Molndal, 431 80 Molndal, Sweden

**Keywords:** Chronic exertional compartment syndrome, Intramuscular pressure, Intracompartmental pressure, Lower limb pain

## Abstract

**Purpose:**

To investigate distributions and identify possible differences in intramuscular pressure (IMP) values at 1 min post-exercise between the four muscle compartments of the lower leg, in patients with exertional leg pain with or without chronic exertional compartment syndrome (CECS).

**Methods:**

A consecutive series of patients seeking orthopaedic consultation for exertional leg pain underwent IMP measurements between 2009 and 2018. The diagnosis of CECS was confirmed (*n* = 442) or ruled out (*n* = 422), based on the patient’s history, clinical examination, and IMP measurements.

**Results:**

The median (range) 1 min post-exercise IMP values in affected compartments in the patients diagnosed with CECS were 33 (25–53) mmHg (deep posterior), 35 (27–54) mmHg (superficial posterior), 40 (26–106) mmHg (lateral), and 47 (24–120) mmHg (anterior). In patients with no CECS, the median (range) 1 min post-exercise IMP values in the compartments were 12 (2–28) mmHg (deep posterior), 12 (2–27) mmHg (superficial posterior), 14 (2–26) mmHg (lateral), and 18 (4–34) mmHg (anterior). The IMP was significantly lower in the lateral and both posterior compartments than in the anterior compartment in both patients diagnosed with CECS and patients without CECS.

**Conclusion:**

The study demonstrates significantly lower IMP values in the posterior and lateral compartments compared to the anterior compartments. These findings suggest a lowering of the IMP 1 min post-exercise cut-off value for diagnosing CECS in the lateral and both posterior compartments, which may lead to improved treatment of patients with suspected CECS in the lower leg.

**Level of evidence:**

Level II.

**Electronic supplementary material:**

The online version of this article (10.1007/s00167-020-06143-w) contains supplementary material, which is available to authorized users.

## Introduction

Chronic exertional compartment syndrome (CECS) causes exercise-induced lower leg pain, which is most commonly experienced by sportsmen and women [8]. Most patients with CECS have a high level of physical activity, with running being the most common individual sport activity and soccer being the most common team activity [4]. The true incidence of CECS in the general population is difficult to determine, as many people tend to modify their activities to decrease the symptoms and do not seek health care [26]. Also, the diagnosis may be missed by clinicians since these patients are asymptomatic at rest and show minimal findings at physical examination [10]. Among a range of distinct entities causing exercise-induced leg pain CECS is one of the most common [19]. The differential diagnosis includes medial tibial stress syndrome, stress fractures, popliteal artery entrapment syndrome, and nerve entrapment syndromes [2]. In a retrospective review of 150 athletes with exercise-induced leg pain, the prevalence of CECS in the lower legs was reported to be 33% [3]. Patients with CECS often have long-standing symptoms, up to several years, before being diagnosed [22]. Further, bilateral symptoms are common, and occur in 67–95% of the patients [12, 22, 24].

CECS is characterized by pain triggered by exertion, attributable to increased intramuscular pressure (IMP) and reduced tissue perfusion within the muscle compartment [20, 27]. Apart from pain, patients with CECS experience muscle tightness and impaired muscle function induced by different types of exercises [1]. There are four distinct muscle compartments in the lower leg: anterior, lateral, superficial posterior, and deep posterior. All four compartments are associated with CECS, but the anterior compartment is the most commonly involved [4]. The treatment for CECS is fasciotomy of the affected compartments.

The pathophysiology of CECS is not fully understood; nor is the relationship between increased IMP and pain. In healthy subjects, IMP increases during exercise and returns to normal on cessation of the activity [22]. In patients with CECS, however, the increase in IMP in the affected compartment becomes abnormally high during exercise and the time for the pressure to return to normal after activity is prolonged, leading to reduced microvascular flow [18, 20]. The abnormally elevated IMP may be related to increased muscle volume, aggravated by a muscle expansion of up to 20% during exercise, in combination with tightness of the fascia [16]. It has been suggested that the pain is caused by traction of the fascia, due to increased IMP, resulting in the compression of sensory nerve endings [1].

To diagnose CECS in patients with exercise-induced leg pain, objective IMP measurements of the lower leg, performed after an exercise test that elicits the pain, are regarded as the gold standard. The IMP criteria for diagnosing CECS are usually based on the Pedowitz diagnostic criteria from 1990, with the IMP cut-off values for all muscle compartments being set at ≥ 15 mmHg pre-exercise, ≥ 30 mmHg at 1 min post-exercise, or ≥ 20 mmHg at 5 min post-exercise [11]. Currently, there is no consensus as to how best to perform or evaluate the IMP measurements in suspected CECS patients, i.e. regarding the symptom-provoking exercise protocol, the timing of IMP measurements in relation to exercise, and the exact IMP cut-off values [15, 17, 23].

One of the challenges in diagnosing CECS, is to consider the anatomical differences in the four muscular compartments of the lower leg. Our current knowledge of post-exercise IMP values are mostly based on measurements of the anterior compartment, and studies of IMP values in all four compartments in larger cohorts of patients with exertional lower leg pain are limited [4]. Comparing post-exercise IMP values between the different compartments in the lower leg may challenge or confirm the most commonly used diagnostic criteria for CECS, and thereby contribute to the accurate treatment of patients with exercise-induced leg pain.

The aim of the present study was to investigate distributions and identify possible differences in IMP values at 1 min post-exercise between the four muscle compartments of the lower leg, in a large cohort of patients with lower leg pain during exercise who were diagnosed to have CECS or not.

## Material and methods

The study protocol was approved by the Regional Ethics Committee in Gothenburg, Sweden (ID number 589-18).

The study population was a consecutive series of 864 patients (486 women and 378 men) who were referred to the Department of Orthopaedics**,** between May 2009 and December 2018 for evaluation of exertional lower leg pain. The patients underwent IMP measurements as part of the consultation. The median age of the overall cohort of patients was 26.3 (range 11–82) years and the median BMI was 24.2 (range 16.6–41.3).

The diagnosis of CECS was either confirmed or ruled out based on the patient’s history, clinical examination, and invasive measurement of IMP following an exercise test. The diagnostic criteria used for CECS included: (1) exercise-induced leg pain and possible impaired muscle function with reversal of symptoms at rest; (2) swelling and/or tenderness over the affected compartment immediately after exercise; (3) IMP ≥ 30 mmHg at 1 min post-exercise and/or IMP ≥ 20 mmHg at 5 min post-exercise. All three criteria were required for the diagnosis of CECS in the anterior, lateral and superficial posterior compartment. The deep posterior compartment was not accessible for evaluation of swelling and more difficult to access for evaluation of tenderness.

### Clinical visits

At the clinical appointment, the medical history was collected and a clinical examination was performed by one of two orthopaedic surgeons, both of whom had several years of experience of the patient group. The lower limbs, including the hip and knee joints, were physically examined. All patients underwent an exercise test aimed at inducing their symptoms, which was followed by measurements of IMP in the symptomatic compartments of their leg(s) at 1 min post-exercise.

The exercise test was individualized and aimed at eliciting the pain that the patient experienced during physical activity, and led up to the referral to the specialist clinic. A typical test started with running on a treadmill, followed by repeated dorsiflexion of the ankle and heel raise in a standing position, alternating between straight and flexed knees. The exercise test was terminated when pain hindered the patient from continuing to perform the activity.

### IMP measurements

To measure IMP, a micro-capillary infusion system (Hemo 4; Siemens, Erlangen, Germany) and a monitor (SC9000; Siemens, Gothenburg, Sweden) were used [9, 14, 21]. The measurements were started 1 min after exercise, with the patient in supine position and the legs extended. External pressure was avoided by using elastic soft supports (ESWELL, Simonsen & Well, Denmark) under the heel and the knee. The IMP needle was connected to a transducer line (length 150 cm) filled with saline, which was linked to the pressure recording system before insertion. To avoid occlusion of the IMP needle tip, the skin over the symptomatic compartment was first penetrated using a separate needle, 1.2 mm in diameter, which was withdrawn before insertion of an 18-gauge (1.2 × 50 mm) IMP needle with four side-holes at its tip. This was inserted into the symptomatic muscle compartment at a 30° angle to the long axis of the leg in a distal direction. At the beginning of the measurements, a slow (0.2 mL/h) infusion of 0.9% saline was maintained through the system and out of the tip of the needle, to maintain the bulging of the fluid at the tip of the IMP needle. The tip of the IMP needle and the transducer were placed at heart level to minimize hydrostatic artefacts. The pressure recording system was calibrated before and after each measurement.

IMP values were measured in the clinically symptomatic compartments of the lower leg, where the pain was localized and/or the muscles were tight on palpation. The 1 min post-exercise IMP values are presented in the present study. When multiple compartments were measured, the most symptomatic compartment was measured first. In patients with bilateral symptoms, only the most symptomatic leg was measured. The 5 min post-exercise IMP values were recorded in patients whose 1 min post-exercise IMP values were between 20 mmHg and 29 mmHg, however only obtained from a small number of patients, and not presented in this study. Measurements of the distance between the fascia and the tip of the IMP needle in the anterior compartment were performed under ultrasound guidance using a linear probe (L10-5, Acuson CV70; Siemens) and performed in 131 patients (68 CECS and 63 non-CECS) and presented in a previous publication; no correlations between the depth of the IMP needle and the IMP measured were found [13].

### Statistical analysis

The IMP data from the four compartments were not normally distributed in patients with CECS, but they were normally distributed in patients without CECS. Results are presented as median and range. Mean IMP values (with SD) in patients without CECS were calculated to determine cut-off values for each compartment in diagnosing CECS. Frequency counts and percentages have been used for categorical variables. Correlations are given with Pearson’s *r *value. Continuous variables between groups were compared using the Mann–Whitney *U* test, and Pearson’s Chi-square test was used for categorical variables. The IMP values for the four compartments in patients with CECS and those without CECS were compared using the Kruskal–Wallis test. Significance was set at *p* < 0.05. Analyses were performed using IBM SPSS software version 26 (IBM Corp., Armonk, NY).

## Results

### Differences in characteristics of patients with CECS and those without CECS

There were significantly more male patients in the CECS group than in the group of patients where the diagnosis was ruled out. The patients with CECS were significantly younger and their BMI was significantly higher. A higher proportion, more than two-thirds, of the patients with CECS had experienced symptoms for ≥ 24 months, as compared to less than three-fifths of those without CECS. Furthermore, a higher proportion of the CECS patients reported having bilateral symptoms (Table 1).

**Table 1:** Characteristics of the patients with chronic exertional compartment syndrome (CECS) and those without CECS (non-CECS)

	CECS	Non-CECS	*p *value
	*n* = 442	*n* = 422	
Median age (range)	25 (13–82)	28 (11–75)	0.002
Males	236 (53%)	142 (34%)	< 0.001
Median BMI (range)^a^	24.9 (18–41)	23.4 (17–36)	< 0.001
Symptoms ≥ 24 months	307 (69%)	247 (59%)	< 0.001
Bilateral symptoms	382 (86%)	294 (70%)	< 0.001

^a^*n* = 815 (418 CECS and 397 non-CECS)

### Number of affected compartments in patients with CECS

The numbers of affected compartments in patients who were diagnosed with CECS, based on the currently used diagnostic criteria, are presented in Table 2. Isolated CECS of the anterior compartment was the most common diagnosis (64%), followed by CECS of both the anterior and lateral compartments (21%).

**Table 2:** Number of isolated or combined compartments affected in patients with chronic exertional compartment syndrome (CECS)

Affected compartments (*n* = 630)	No. of patient (*n* = 442)	Percentage of patients with CECS
Isolated anterior	281	63.5
Isolated lateral	9	2
Isolated superficial posterior	3	0.7
Isolated deep posterior	9	2
Anterior and lateral	91	20.6
Anterior, lateral, and deep posterior	3	0.7
Anterior, lateral, and superficial posterior	3	0.7
Anterior and deep posterior	5	1.1
Anterior and superficial posterior	3	0.7
Anterior, superficial, and deep posterior	6	1.4
Superficial and deep posterior	11	2.5
All compartments	18	4.1

### Variations in median IMP values between muscle compartments of the lower leg in patients with CECS and those without CECS

The 1 min post-exercise IMP values in the affected compartments in patients with CECS and in all compartments measured in patients without CECS are presented in Table 3. There were 21 patients diagnosed with CECS who had a 1 min post-exercise IMP < 30 mmHg. The CECS diagnosis of these patients was based on IMP values ≥ 20 mmHg 5 min post-exercise, in addition to the patient’s history and the clinical findings.

**Table 3:** Intramuscular pressure (IMP) in patients with chronic exertional compartment syndrome (CECS) and without CECS (non-CECS)

Compartment	Median IMP (mmHg)		Interquartile range IMP (mmHg)		Min–Max IMP (mmHg)	
	CECS (*n* = 442)	Non-CECS (*n* = 422)	CECS	Non-CECS	CECS	Non-CECS
Anterior	47	18	37–60	13–25	24–120	4–34
Lateral	40	14	33–55	8–18	26–106	2–26
Superficial posterior	35	12	31–42	10–18	27–54	2–27
Deep posterior	33	12	30–38	8–17	25–53	2–28

The highest IMP values in the anterior and lateral compartments in patients with CECS were 120 mmHg and 106 mmHg, respectively. In the superficial and deep posterior compartments of patients with CECS, the highest IMP values were just above 50 mmHg. The IMP values in the anterior compartment were 50 mmHg or higher in 15 (83%) of the patients with CECS in all compartments.

**Fig. 1:**
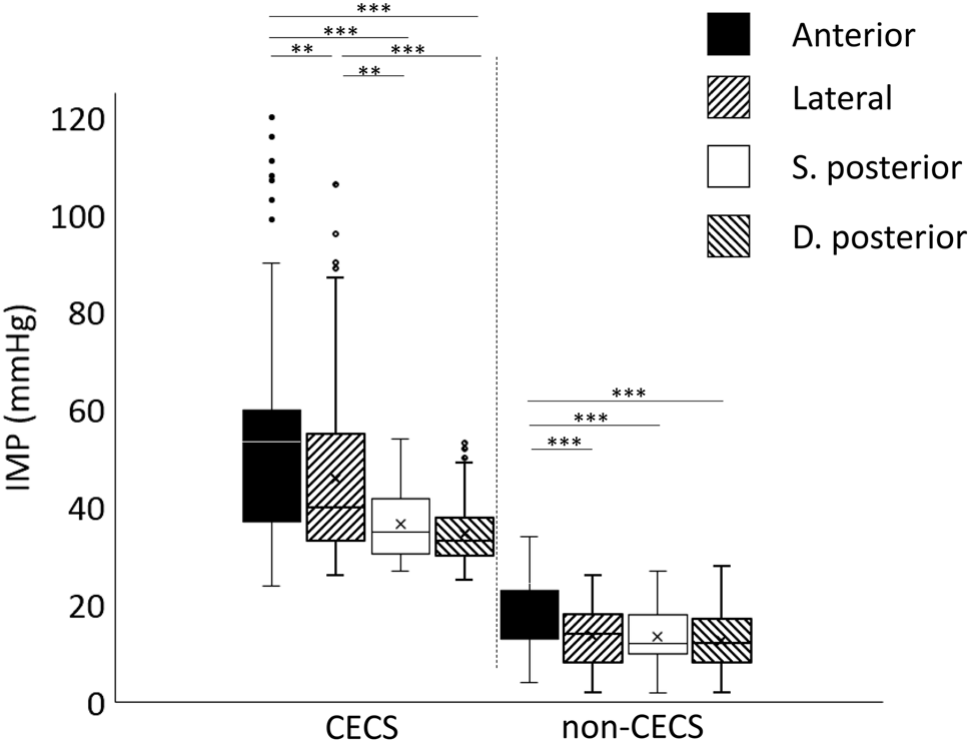
Differences in 1 min post-exercise intramuscular pressure (IMP) values in the four lower leg compartments. 1 min post-exercise IMP values in the affected four lower leg muscle compartments in 442 patients diagnosed with chronic exertional compartment syndrome (CECS) and in all compartments measured in 422 patients without CECS (non-CECS). The IMP values for the anterior, lateral, superficial posterior, and deep posterior compartments were obtained by invasive measurements with a micro-capillary infusion system and compared using the Kruskal–Wallis test. The boxes represent the interquartile range and the whiskers represent the highest and lowest values. Outliers are indicated by circles. *S. posterior*, superficial posterior; *D. posterior*, deep posterior. *p < 0.05, **p < 0.01, ***p < 0.001

The comparison between IMP levels in the four muscle compartments is presented in Fig. 1. In affected compartments in patients with CECS, the median IMP values in the superficial posterior and deep posterior compartments were significantly lower than in the affected anterior compartments, median difference more than 10 mmHg (*p* < 0.001). Furthermore, the median IMP values were significantly lower in the affected superficial posterior compartments (*p* = 0.026) and deep posterior compartments (*p* < 0.001) than in the lateral compartments. The median IMP was also significantly lower in the affected lateral compartments than in the affected anterior compartments (*p* = 0.005). In patients without CECS, the median IMP values in the lateral, superficial posterior, and deep posterior compartments were significantly lower than the median IMP in the anterior compartment (*p* < 0.001).

### Distribution of 1 min post-exercise IMP for compartments in patients with CECS and those without CECS

**Fig. 2:**
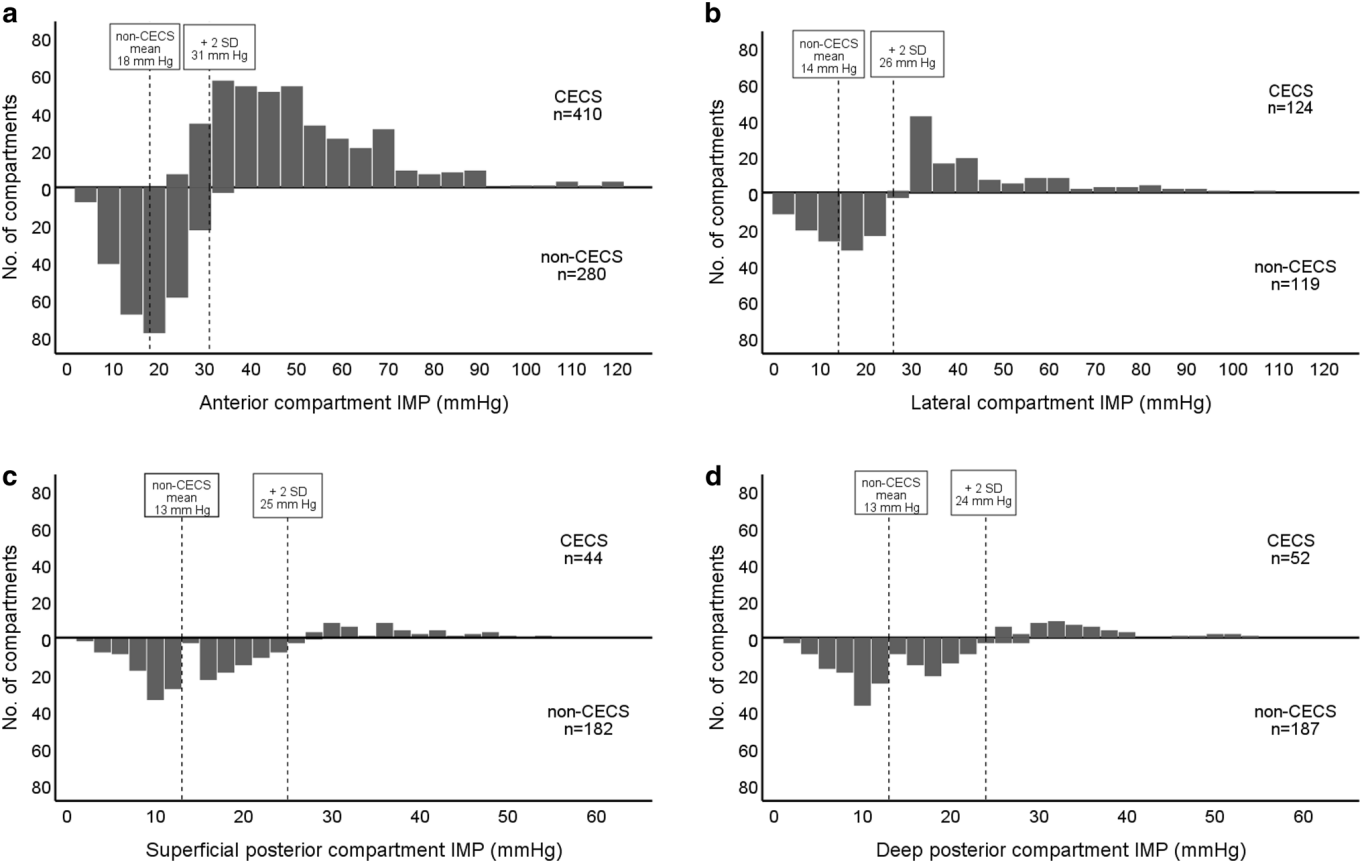
Distribution of 1 min post-exercise intramuscular pressure (IMP) values in the four lower leg compartments. Distribution of IMP values 1 min post-exercise in affected anterior compartments (**a**), lateral compartments (**b**), superficial posterior compartments (**c**), and deep posterior compartments (**d**) of 442 patients with chronic exertional compartment syndrome (CECS) and in all compartments measured in 422 patients without CECS (non-CECS). In each histogram, the mean IMP value and the mean plus two standard deviations (2 SD) are shown for the patients without CECS. Note the different scales on the x-axis between** a**,** b** and** c**,** d**

The distributions of the 1 min post-exercise IMP values for the affected compartments in patients with CECS and in patients without CECS are presented in Fig. 2. The mean IMP value and the mean plus two standard deviations (2 SD) are shown for the compartments in the patients for whom the CECS diagnosis was ruled out.

## Discussion

The main finding of the present cohort study of CECS patients was that the median IMP values in affected posterior and lateral compartments were significantly lower than that in affected anterior compartments. Furthermore, the median IMP values in patients with no CECS showed a similar variation in distribution regarding the four compartments to that seen in the CECS patients, with the highest IMP values in the anterior compartment. These findings suggest an adjustment of the IMP 1 min post-exercise cut-off value for diagnosing CECS in both of the posterior compartments and the lateral compartment.

In the present cohort, isolated CECS in the anterior compartment was the most common diagnosis (64%)—as reported in earlier studies [1, 4]. In the present study, the highest measured IMP values were obtained in the anterior compartments. The mean IMP 1 min post-exercise value plus 2 SD, recorded in 280 anterior compartments in patients with no CECS, was 31 mmHg. The currently used IMP 1 min post-exercise cut-off value of 30 mmHg for CECS appears to fit well with the distribution curves of the IMP measurements for the anterior compartments in the present study.

The finding of variation in median IMP values between the different compartments in the lower leg—both for affected compartments in patients with CECS and for compartments in patients with no CECS—may be explained by anatomical differences. The diagnosis of CECS is usually based on patient history combined with the Pedowitz diagnostic criteria, in which the cut-off IMP values of ≥ 30 mmHg at 1 min post-exercise or ≥ 20 mmHg at 5 min post-exercise for CECS, have been suggested to be the same for all four muscle compartments of the lower leg. The suggested cut-off value was based on the total mean IMP value for all four compartments plus 2 SD in patients with no CECS, a total of 210 compartments [11].

In the present study, the IMP values recorded in 182 superficial posterior and 187 deep posterior compartments in patients with no CECS showed a mean IMP 1 min post-exercise plus 2 SD (representing the 95% CI) of 25 mmHg and 24 mmHg, respectively. Based on these findings, the most commonly used IMP 1 min post-exercise cut-off value of 30 mmHg appears to be too high for the posterior compartment, and lower cut-off values might be considered. In addition, the mean 1 min post-exercise IMP plus 2 SD was 26 mmHg based on IMP recordings in 119 lateral compartments of patients with no CECS. This value is also below the currently recommended IMP cut-off value.

The IMP differences between the four compartments both for CECS and non-CECS patients suggest a customization of the cut-off values for IMP measurements**.** Based on the 95% CI for IMP measurements in patients without CECS, we propose a lowering of the IMP 1 min post-exercise cut-off value for diagnosing CECS in both of the posterior compartments and the lateral compartment. The use of a lower cut-off value may improve the possibility of setting a correct diagnosis, and thereby improve the treatment of patients with suspected CECS in the posterior compartments and/or the lateral compartment of the lower leg. However, the clinical effect of such adjustment of the diagnostic cut-off IMP values should be investigated further in studies evaluating the results of fasciotomy for patients with IMP measurements in the lower intervals.

In the present study, there were significantly more male patients in the group with CECS than in the group of patients for whom the diagnosis was ruled out, even though there was a higher proportion of women (56%) in the overall study population. The male predominance in the present study in patients with CECS is supported by the findings of earlier studies [6, 11, 13]. However, in some more recent studies, the opposite—a higher proportion of female patients with CECS—has also been reported [4, 26]. The median age of the patients with CECS was significantly lower and their symptoms were more commonly bilateral than in the patients with no CECS. Male gender, young age, and bilateral symptoms have been put forward as independent predictive factors in a recent study of 1411 individuals with exertional lower leg pain [5].

A higher proportion of the patients with CECS in the present study had had their symptoms for ≥ 24 months compared to the patients in whom the diagnosis was ruled out. The finding of long-standing symptoms is in line with the results of a previous study, in which the mean duration of symptoms in patients with CECS was 24 months [22]. Long-standing symptoms before diagnosis may be due to a delay by the medical profession to diagnose the CECS. With incorrect diagnosis patients may undergo unsuccessful treatments and athletes may give up their sports routine [10]. Further, repeated IMP measurements to confirm the diagnosis of CECS may be needed in some patients. In a recent study, CECS was diagnosed in 3 out of 15 patients undergoing a re-IMP measurement for an ongoing suspicion of CECS despite a normal first IMP measurement [25].

The limitations of the present study include recruitment of patients with clinical symptoms of exercise-induced leg pain only, and the lack of a healthy control group. As measurement of IMP is an invasive method with associated pain and a risk for infections, there are ethical difficulties to perform such study in a large cohort. Moreover, IMP values were measured only in symptomatic compartments, due to the risk of complications associated with invasive measurements [7]. For measurements from multiple compartments, the most symptomatic compartment was measured first. The sequence of the measuring procedure unfortunately does not allow all the compartments to be measured simultaneously, why a slight pressure decline might have occurred for the last measured compartment(s) when several compartments were measured. This delay between compartments potentially could have contributed to the lower values in the lateral and posterior compartments. However, the measurement takes approximately 15 s for each compartment. In CECS patients the time for the increase in IMP in the affected compartment(s) to return to normal values after activity is prolonged [18, 20], why this delay cannot explain the overall results.

One strength of the present study was the inclusion of such a large cohort of patients with exertional lower leg pain, all clinically examined and underwent IMP measurement in a standardized manner and were examined by one of two experienced clinicians at the same clinic.

The findings from the present study suggests adjustments of the IMP cut-off values which may improve the possibility of identifying patients with CECS in the posterior compartments and/or the lateral compartment of the lower leg, and thereby could improve the treatment strategy for these patients.

## Conclusion

One minute post-exercise IMP values in patients diagnosed with CECS are significantly lower in affected posterior and lateral compartments compared to affected anterior compartments. Based on these findings, a lowering of the IMP 1 min post-exercise cut-off value for diagnosing CECS in the lateral compartment and both posterior compartments could be suggested.

## Electronic supplementary material

Below is the link to the electronic supplementary material.

Supplementary file 1 (PPTX 38 kb)

## Abbreviations

CECS: Chronic exertional compartment syndrome
Non-CECS: Patients without CECS
IMP: Intramuscular pressure

## References

[1] Blackman PG (2000). A review of chronic exertional compartment syndrome in the lower leg. Med Sci Sports Exerc.

[2] Burrus MT, Werner BC, Starman JS, Gwathmey FW, Carson EW, Wilder RP (2015). Chronic leg pain in athletes. Am J Sports Med.

[3] Clanton TO, Solcher BW (1994). Chronic leg pain in the athlete. Clin Sports Med.

[4] Davis DE, Raikin S, Garras DN, Vitanzo P, Labrador H, Espandar R (2013). Characteristics of patients with chronic exertional compartment syndrome. Foot Ankle Int.

[5] de Bruijn JA, van Zantvoort APM, van Klaveren D, Winkes MB, van der Cruijsen-Raaijmakers M, Hoogeveen AR (2018). Factors predicting lower leg chronic exertional compartment syndrome in a large population. Int J Sports Med.

[6] Detmer DE, Sharpe K, Sufit RL, Girdley FM (1985). Chronic compartment syndrome: diagnosis, management, and outcomes. Am J Sports Med.

[7] Hislop M, Batt ME (2011). Chronic exertional compartment syndrome testing: a minimalist approach. Br J Sports Med.

[8] Lohrer H, Malliaropoulos N, Korakakis V, Padhiar N (2019). Exercise-induced leg pain in athletes: diagnostic, assessment, and management strategies. Phys Sports Med..

[9] Nilsson A, Zhang Q, Styf J (2014). Using the amplitude of pulse-synchronous intramuscular pressure oscillations when diagnosing chronic anterior compartment syndrome. Orthop J Sports Med.

[10] Paik RS, Pepple DA, Hutchinson MR (2013). Chronic exertional compartment syndrome. BMJ.

[11] Pedowitz RA, Hargens AR, Mubarak SJ, Gershuni DH (1990). Modified criteria for the objective diagnosis of chronic compartment syndrome of the leg. Am J Sports Med.

[12] Reneman RS (1975). The anterior and the lateral compartmental syndrome of the leg due to intensive use of muscles. Clin Orthop Relat Res.

[13] Rennerfelt K, Zhang Q, Karlsson J, Styf J (2016). Changes in muscle oxygen saturation have low sensitivity in diagnosing chronic anterior compartment syndrome of the leg. J Bone Joint Surg Am.

[14] Rennerfelt K, Zhang Q, Karlsson J, Styf J (2018). Patient pain drawing is a valuable instrument in assessing the causes of exercise-induced leg pain. BMJ Open Sport Exerc Med.

[15] Roberts A, Franklyn-Miller A (2012). The validity of the diagnostic criteria used in chronic exertional compartment syndrome: a systematic review. Scand J Med Sci Sports.

[16] Rorabeck C, Macnab I (1975). The pathophysiology of the anterior tibial compartmental syndrome. Clin Orthop Relat Res.

[17] Roscoe D, Roberts AJ, Hulse D (2015). Intramuscular compartment pressure measurement in chronic exertional compartment syndrome: new and improved diagnostic criteria. Am J Sports Med.

[18] Sindhu K, Cohen B, Gil JA, Blood T, Owens BD (2019). Chronic exertional compartment syndrome of the forearm. Phys Sportsmed.

[19] Styf J (1988). Diagnosis of exercise-induced pain in the anterior aspect of the lower leg. Am J Sports Med.

[20] Styf J, Korner L, Suurkula M (1987). Intramuscular pressure and muscle blood flow during exercise in chronic compartment syndrome. J Bone Joint Surg Br.

[21] Styf JR, Körner LM (1986). Microcapillary infusion technique for measurement of intramuscular pressure during exercise. Clin Orthop Relat Res.

[22] Turnipseed WD (2002). Diagnosis and management of chronic compartment syndrome. Surgery.

[23] Tzortziou V, Maffulli N, Padhiar N (2006). Diagnosis and management of chronic exertional compartment syndrome (CECS) in the United Kingdom. Clin J Sport Med.

[24] van Zantvoort AP, de Bruijn JA, Winkes MB, Dielemans JP, van der Cruijsen-Raaijmakers M, Hoogeveen AR (2015). Isolated chronic exertional compartment syndrome of the lateral lower leg: a case series. Orthop J Sports Med.

[25] van Zantvoort APM, de Bruijn JA, Winkes MB, Hoogeveen AR, Teijink JAW, Scheltinga MR (2017). Role of repeat muscle compartment pressure measurements in chronic exertional compartment syndrome of the lower leg. Orthop J Sports Med.

[26] Waterman BR, Liu J, Newcomb R, Schoenfeld AJ, Orr JD, Belmont PJ (2013). Risk factors for chronic exertional compartment syndrome in a physically active military population. Am J Sports Med.

[27] Zhang Q, Jonasson C, Styf J (2011). Simultaneous intramuscular pressure and surface electromyography measurement in diagnosing the chronic compartment syndrome. Scand J Med Sci Sports.

