# Supplementary material for: Significantly lower intramuscular pressure in the posterior and lateral compartments compared with the anterior compartment suggests alterations of the diagnostic criteria for chronic exertional compartment syndrome in the lower leg
Source: Knee Surg Sports Traumatol Arthrosc. 2020 Jul 8;29(4):1332–9. doi: 10.1007/s00167-020-06143-w (PMC7973917; doi:10.1007/s00167-020-06143-w)
Supplement: Supplementary file 1 — (PPTX 38 kb) [file 167_2020_6143_MOESM1_ESM.pptx]

## Slide 1
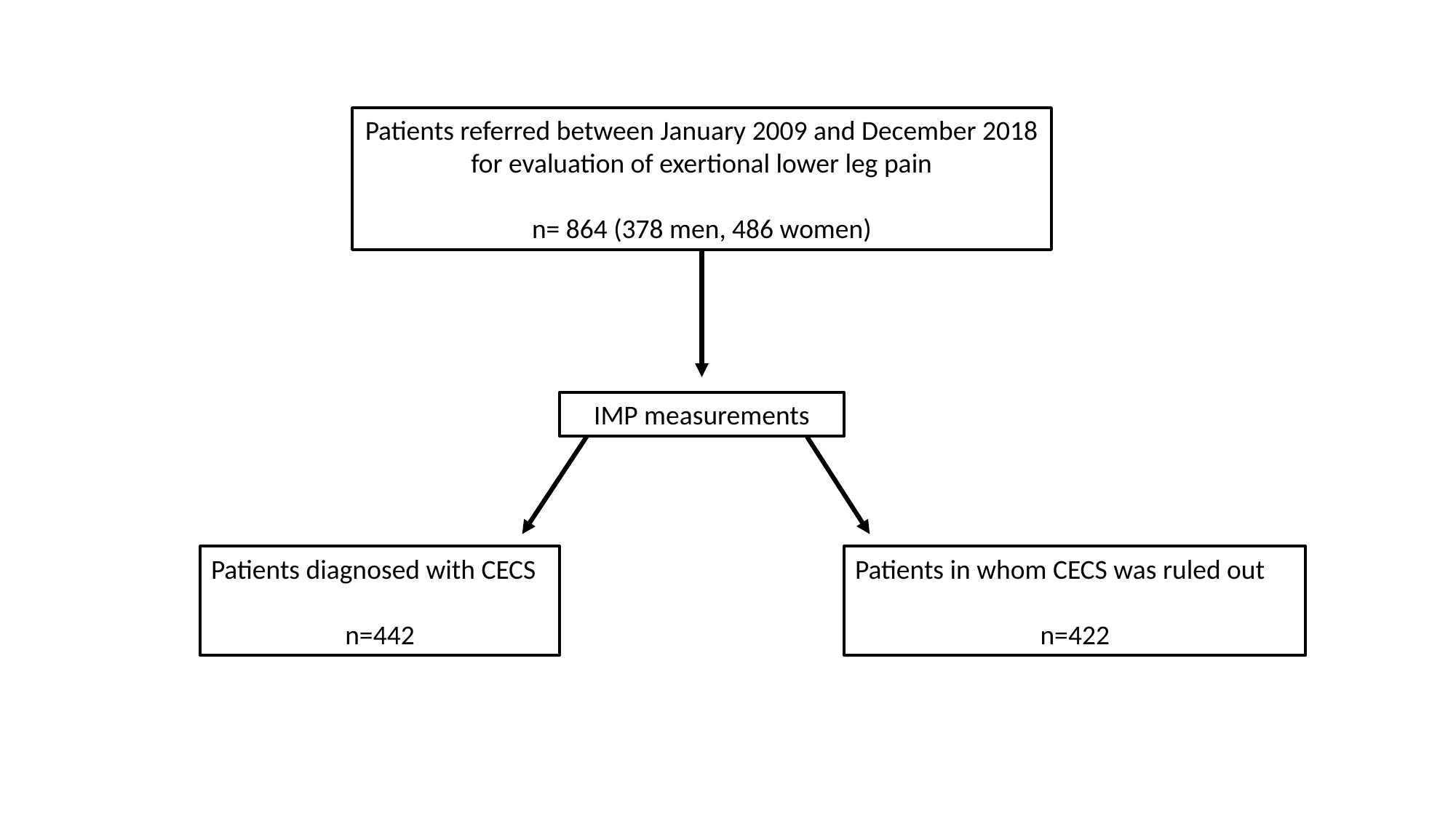

Patients referred between January 2009 and December 2018 for evaluation of exertional lower leg pain
n= 864 (378 men, 486 women)
IMP measurements
Patients diagnosed with CECS
n=442
Patients in whom CECS was ruled out
n=422
